# Diagnostic and Therapeutic Approach to Children and Adolescents with Obstructive Sleep Apnea Syndrome (OSA): Recommendations in Emilia-Romagna Region, Italy

**DOI:** 10.3390/life12050739

**Published:** 2022-05-16

**Authors:** Susanna Esposito, Giampiero Ricci, Riccardo Gobbi, Claudio Vicini, Fabio Caramelli, Silvia Pizzi, Agatina Fadda, Salvatore Ferro, Giuseppe Plazzi

**Affiliations:** 1 Pediatric Clinic, Department of Medicine and Surgery, University Hospital, University of Parma, 43126 Parma, Italy; 2 Pediatric Unit, IRCCS Azienda Ospedaliero-Universitaria di Bologna, 40138 Bologna, Italy; giampiero.ricci@unibo.it; 3 Head-Neck and Oral Surgery Unit, Department of Head-Neck Surgery, Otolaryngology, Morgagni Piertoni Hospital, 47121 Forlì, Italy; riccardo.gobbi@auslromagna.it (R.G.); claudio@claudiovicini.com (C.V.); 4 Pediatric Intensive Care Unit, IRCCS Azienda Ospedaliero-Universitaria di Bologna, 40138 Bologna, Italy; fabio.caramelli@aosp.bo.it; 5 Dentistry Unit, Department of Medicine and Surgery, Centro Universitario di Odontoiatria, University of Parma, 43126 Parma, Italy; s.pizzi@unipr.it; 6 Direzione Generale Cura della Persona, Salute e Welfare, Emilia-Romagna Region, 40128 Bologna, Italy; agatina.fadda@regione.emilia-romagna.it (A.F.); salvatore.ferro@regione.emilia-romagna.it (S.F.); 7 IRCCS Istituto delle Scienze Neurologiche di Bologna, 40139 Bologna, Italy; giuseppe.plazzi@unibo.it; 8 Department of Biomedical, Metabolic and Neural Sciences, University of Modena and Reggio Emilia, 41125 Modena, Italy

**Keywords:** breathing disorder, hypoventilation, obstructive sleep apnea syndrome, OSA, paediatrics

## Abstract

Obstructive sleep apnoea syndrome (OSA) in paediatrics is a rather frequent pathology caused by pathophysiological alterations leading to partial and prolonged obstruction (hypoventilation) and/or intermittent partial (hypopnoea) or complete (apnoea) obstruction of the upper airways. Paediatric OSA is characterised by daytime and night-time symptoms. Unfortunately, there are few data on shared diagnostic-therapeutic pathways that address OSA with a multidisciplinary approach in paediatric age. This document summarizes recommendations from the Emilia-Romagna Region, Italy, developed in order to provide the most appropriate tools for a multidisciplinary approach in the diagnosis, treatment and care of paediatric patients with OSA. The multidisciplinary group of experts distinguished two different ‘step’ pathways, depending on the age group considered (i.e., under or over two years). In most cases, these pathways can be carried out by the primary care paediatrician, who represents the first filter for approaching the problem. For this reason, it is essential that the primary care paediatrician receives adequate training on how to formulate the diagnostic suspicion of OSA and on what criteria to use to select patients to be sent to the hospital centre. The relationship between the paediatrician of the patient and her/his parents must see a synergy of behaviour between the various players in order to avoid uncertainty about the diagnostic and therapeutic decisions as well as the follow-up phase. The definition and evaluation of the organizational process and outcome indicators of the developed flow-chart, and the impact of its implementation will remain fundamental.

## 1. Introduction

Obstructive sleep apnoea syndrome (OSA) in paediatrics is a rather frequent pathology caused by pathophysiological alterations leading to partial and prolonged obstruction (hypoventilation) and/or intermittent partial (hypopnoea) or complete (apnoea) obstruction of the upper airways [1,2,3]. This condition causes an absence of airflow despite continuous respiratory effort and is usually associated with reduced peripheral oxygen saturation and/or hypercapnia. Children with OSA have a respiratory effort that tends to overcome resistance in the upper airway, which is normally a trigger for producing arousals. Since obstructive events are mainly typical of REM sleep, which is characterised by muscular hypotonia, children with OSA usually have more arousals than those without OSA [1,2,3].

Paediatric OSA is characterised by daytime and night-time symptoms [4,5]. Diurnal symptoms include shortness of breath, irritability, nasal voice, chronic rhinitis, morning headache, poor school concentration, growth retardation and, more rarely, arterial hypertension and cardiac changes. During the night, snoring, pauses in breathing during sleep, shortness of breath, changes in hearth rate, changes in skin colour, choking sensation, fear or nocturnal agitation, abnormal sleeping positions, paradoxical movements of the chest and abdomen, intense sweating, insomnia, nocturnal enuresis, sleepwalking and/or bruxism are present. If not treated properly, OSA can lead to complications, sometimes serious, even in childhood, mainly due to intermittent hypoxemia [4,5,6]. These can cause a chronic inflammatory state leading to increased production of free radicals and other reactive oxygen species, which are responsible for oxidative stress. In addition to this, the fragmentation of the normal sleep pattern to which the paediatric patient with OSA is exposed deserves special consideration. These consequences have a major impact on cognitive development, especially if OSA is present from the earliest years of life. Several studies have, in fact, shown that untreated OSA patients more often present cognitive or neuropsychological function deficits (i.e., general intelligence, verbal intelligence, executive functions, learning, memory, visuospatial skills, language, mathematical skills, abstract and analytical thinking) [7]. Behavioural alterations, particularly hyperactivity, in younger children, and emotional lability, anxiety and depression are other frequent morbidity factors in paediatric patients with sleep disorders. However, although it is still controversial, some authors demonstrated that early treatment of paediatric OSA can improve the patient’s cognitive ability and school and social performance [7]. Unfortunately, there are few data on shared diagnostic-therapeutic pathways that address OSA with a multidisciplinary approach in paediatric age.

Due to the lack of standardized multidisciplinary protocols on OSAS in paediatric age, this document summarizes recommendations from Emilia-Romagna Region, Italy, developed in order to provide the most appropriate tools for a multidisciplinary approach in the diagnosis, treatment and care of paediatric patients with OSA.

## 2. Epidemiology

OSA can occur throughout childhood, with a peak incidence between the second and sixth years of age [2,3]. Estimates of its prevalence in children are quite variable due to the different inclusion criteria adopted and the different pulse oximetry and polysomnographic parameters used for diagnosis. Currently, the prevalence of OSA in children is thought to be between 2% and 5.7% [2,3]. Obesity, male sex, the degree of severity of OSA and persistent adenotonsillar hypertrophy with mandibular hypoplasia have been shown to be risk factors for the persistence of the disease [2,3].

## 3. Aetiopathogenesis

By far the most frequent cause of OSA in paediatric age, particularly over two years of age, is adenotonsillar hypertrophy [1,2,3]. Other less frequent aetiological factors are excess weight and craniofacial dysmorphisms [8]. On the other hand, children aged <23 months are more prone to obstruction due to anatomical factors (i.e., shape of the face, small airways, forced nasal breathing), pulmonary mechanics (i.e., low residual functional capacity), immature and variable ventilatory control, prevalence of the REM phase of sleep (which can exacerbate obstruction due to reduced muscle tone) and easy collapsibility of the upper airways. In this age group, the most common causes of OSA [9] are:

- Craniofacial anomalies (particularly mandibular hypoplasia, as in the Pierre Robin sequence);
- Genetic syndromes (i.e., achondroplasia, Down’s syndrome, Prader-Willi syndrome);
- Nasal obstruction (i.e., respiratory infections, coana atresia);
- Laryngeal obstruction (i.e., laryngeal malformations, laryngomalacia, vocal cord paralysis);
- Neurological and neuromuscular diseases (i.e., cerebral palsy, mitochondrial diseases, spinal muscular atrophy);
- Gastroesophageal reflux;
- Adenotonsillar hypertrophy after the age of six months.

Comorbidities include pulmonary hypertension, growth retardation, prematurity, behavioural problems, and feeding difficulties.

## 4. Diagnosis

### 4.1. Anamnestic Data

The suspicion of OSA must be raised by means of anamnestic and clinical criteria already during routine visits by the primary care paediatrician. In more details, the anamnestic information that the primary care paediatrician should note is:

- Presence of habitual snoring (three or more nights a week);
- Presence of breathing difficulties during sleep (apnoeas, noisy breathing or gasping);
- Enuresis;
- Preferred position during sleep (sitting or with hyperextended neck); cyanosis;
- Headache on awakening; daytime sleepiness;
- Cognitive and behavioural deficits (Table 1);
- In the first two years of life, poor growth or feeding difficulties; family history of adenotonsillectomy.

The use of clinical and anamnestic questionnaires (Pediatric Sleep Questionaire [10] or Teenager STOP-BANG [11], available as Supplementary Materials S1 and S2) is useful for a more accurate picture in support of the clinic. In children less than two years of age, suspicion of OSA may be advanced by the presence of a previous episode of acute life threatening event (ALTE)/brief resolved unexplained event (BRUE).

### 4.2. Clinical Picture

During the physical examination, particular attention should be paid to the possible presence of:

- Weight loss or gain, obesity;
- Growth deficit;
- Tonsillar hypertrophy;
- Adenoid facies;
- Micro/retrognancy;
- Ogival palate;
- Dental malocclusions;
- Craniofacial malformations (i.e., Pierre Robin sequence, craniostenosis, Apert syndrome, Crouzon syndrome, Treacher Collins syndrome, cleft lip and palate);
- Genetic and metabolic diseases (i.e., down syndrome, Prader-Willi syndrome, mucopolysaccharidosis);
- Infantile cerebral palsy;
- Neuromuscular disease;
- Arterial hypertension;
- Laryngomalacia with inspiratory stridor (in younger children).

Paediatric OSA recognises three phenotypes [8] based on objective examination:

- Classic phenotype, characterised by adenotonsillar hypertrophy with or without dental and skeletal malocclusion;
- Adult phenotype, characterised by obesity associated or not with aspects of the classical phenotype;
- Congenital phenotype, characterised by craniofacial anomalies associated with genetic syndromes (i.e., Pierre Robin sequence, Crouzon syndrome, Apert syndrome, Down syndrome).

### 4.3. Instrumental Examinations

After an accurate clinical and anamnestic assessment, possibly associated with an otorhinolaryngological examination with rhinofibrolaryngoscopy (under sedation in spontaneous breathing in the case of assessment of the lower airways), the diagnosis of OSA must necessarily be confirmed by means of instrumental examinations [12].

The instrumental methods for the diagnosis of OSA in paediatric age [12] are:

1. Night-time pulse oximetry with memory;
2. Night-time cardiorespiratory monitoring (polygraph);
3. Nocturnal polysomnography (PSG), which is the gold standard.

Abbreviated polysomnography (nappolygraphy) during afternoon sleep tends, due to its brevity, is associated with the risk to underestimate the prevalence and severity of OSA and in the case of negativity does not allow the exclusion of OSA [12]. Therefore, it is not a test to be used for the diagnosis of OSA.

Drug induced sleep endoscopy (DISE) is performed following an instrumental diagnosis of OSA and in selected cases to reveal the site and type of respiratory obstruction [12].

Night-time pulse oximetry with memory is carried out at home and is therefore inexpensive and easy to perform. Its positive predictive value is 97% in cases of severe OSA [2,13]. The limitation of this method is the impossibility of diagnosing non-desaturating apneas or hypopneas, which justifies the low sensitivity of the examination. In order to limit this problem as much as possible, it is advisable to use pulse oximeters with a short averaging time (approx. 3 s), which also allow short duration desaturations to be captured. Pulse oximetry can also be affected by motion artefacts, especially in children. To overcome this technological limitation, it is advisable to use pulse oximeters equipped with artefact filtering systems and which allow visualisation of the plethysmographic wave in order to carry out a visual analysis of the events recorded. With these precautions we try to avoid falsely high values of the number of desaturations per hour (oxygen desaturation index (ODI)) which could generate false positives. It should be noted that, despite the limit of low sensitivity, a positive nocturnal saturation test avoids the need for other more complex and costly instrumental examinations, at least in OSA due to adenotonsillar hypertrophy [14]. If pulse oximetry is negative and there are symptoms suggestive of OSA, it is advisable to repeat pulse oximetry in the first instance. If the clinical-instrumental discrepancy persists, other, more complex diagnostic tests are recommended.

Cardiorespiratory monitoring in sleep identifies cardiorespiratory events (central or mixed obstructive apnoeas, hypopnoeas, respiratory periodicity, desaturations, electrocardiographic alterations, paradoxical breathing) occurring in sleep through a polygraphic recording [11]. It does not allow for the assessment of sleep architecture but has the advantage that it can also be performed at the patient’s home. The recording can be made more accurate by adding CO_2_ measurement (end-tidal or transcutaneous) and video recording to the polygraphic system.

Even in paediatrics, PSG represents the diagnostic gold standard for OSA as it allows recording of respiratory events occurring in relation to sleep phases and includes also electroencephalographic, electrooculographic and electromyographic derivations. As with cardiorespiratory monitoring, PSG may include a channel for CO_2_ measurement and video recording [15]. PSG allows the detection of so-called Respiratory Events Related Arousals (RERA), which may be the only manifestation in milder forms of OSA. It is particularly indicated in patients with neuromuscular diseases, craniofacial abnormalities, obesity and in patients who have already undergone adenotonsillectomy in which OSA persists [15]. Furthermore, it should be used before and after the application of the maxillary expander and the application of continuous positive pressure (CPAP) or two-level positive pressure (BiPAP) [15]. The complexity of the method makes standard PSG an examination to be performed only in specialist centres and in selected cases.

DISE provides additional information on the site(s) and patterns of upper airway narrowing and obstruction in OSA [16]. It is performed in selected patients in whom this additional information regarding upper airway dynamics (VADS) is deemed useful. This method is an added value for the surgical or conservative therapeutic outcome [continuous positive pressure (C-PAP) or oral appliance (OA)] in strictly selected patients. The directions to DISE [16] are:

- Residual OSA after initial surgical treatment;
- OSA associated with syndromic disorders;
- Severe OSA and inconsistent ENT findings (adenoidal grade G1 or G2 and tonsillar grade G1 or G2).

All subjects who are candidates for DISE must first undergo an instrumental examination that is diagnostic for OSA. DISE is carried out at 2nd level centres in Phase 3 as an in-patient procedure.

## 5. Classification of OSA

The classification of OSA varies depending on the method used. The McGill oximetry score is frequently used to classify OSA based on pulse oximetry and consists of assessing the number of desaturations below 90%, 85%, and 80% of peripheral oxygen saturation (SpO2) and their organisation into clusters over a sleep duration of between 10 and 30 min. By definition, the cluster must include at least five desaturation events of at least 4% above the mean SpO2 [17].

A positive saturation test for OSA must include at least three clusters of desaturations. Mild forms of OSA are those with at least three desaturations below 90%, medium forms are those that also include three or more desaturations below 85% and severe forms are those that also include three or more desaturations below 80% [17]. Alterations in saturation that do not fall within these parameters are considered inconclusive and therefore require a PSG as the risk of false negatives is high [17].

According to the statements provided by the European Respiratory Society, the polysomnographic classification of OSA is based on the number of apneas-hypopneas per hour (Apnea Hypopnea Index, AHI) [2,3]. In children, mild forms are considered those in which the AHI is between 1 and 5 events/h with SpO2 nadir between 86% and 91%, medium forms are those with AHI between 5 and 10 events/h and nadir between 76% and 85%, and severe forms are those with AHI greater than 10 events/h and nadir less than 75% [2,3].

## 6. Therapy

Paediatric OSA is a multifactorial disease and therefore requires a multidisciplinary approach involving the family paediatrician, the paediatrician-pneumologist specialising in sleep-disordered breathing, the otorhinolaryngologist, the child neuropsychiatrist/neurologist (hospital and/or territorial), the orthodontist and other specialists (maxillofacial surgeon, cardiologist, anaesthetist) depending on the patient’s clinical picture. Although watchful waiting as therapeutic approach can be useful in the majority of the cases, early treatment is essential in selected patients to improve the child’s long-term outcome, especially when cognitive and/or behavioural problems coexist. Treatment of OSA has been shown to be associated with improvements in behaviour, attention and social relationships.

### 6.1. Medical Therapy

It is based on the use of topical nasal corticosteroids [17,18] in combination or not with oral antileukotrienes [19], which can be used in the treatment of mild forms, in residual forms after adenotonsillectomy (AT) surgery or as standby therapy before AT, orthodontic surgery or CPAP application.

### 6.2. Surgical Therapy

In the treatment of OSA due to adenotonsillar hypertrophy, AT is the first choice [20]. The surgical technique can be that of extracapsular, or classical AT or intracapsular AT/tonsillotomy with various technologies (i.e., debrider, plasma scalpel). Both techniques are considered effective in the literature with weak evidence of less bleeding and postoperative pain for intracapsular techniques [20].

The estimated effectiveness of surgical treatment ranges from 70% to 100% of cases [20]. The age of less than three years, obesity, the presence of structural or functional alterations of the upper airways (craniofacial syndromes, neuromuscular pathologies), the presence of cardiac co-morbidities, concomitant infections of the upper airways, are all conditions that must lead to careful post-operative in-patient monitoring [14]. The minimum age for performing adenoidectomy is three months, and for AT six months. Other organic alterations have the possibility of surgical correction, for example coanal atresia, laryngomalacia, labiopalatoschisis and craniofacial malformations (mid-facial hypoplasia and mandibular-retrognathic hypoplasia) [20]. In these cases, laser supraglottoplasty, endoscopic correction of coanal atresia, tracheostomy, or maxillofacial surgery are effective and must be planned and performed in a multidisciplinary and dedicated care setting. In patients with craniofacial malformations, surgery is aimed at widening the space of the upper airway. In this group of patients, the surgical indication and timing of surgery should be discussed in a multidisciplinary setting.

### 6.3. Orthodontic Therapy

It is aimed at widening the hard palate through the application of a fixed orthodontic appliance with an active phase of approximately two to four months followed by a stabilisation phase of at least 6 months to reduce the risk of recurrence. It is indicated in children with transverse contraction of the upper jaw and dental malocclusion [21].

Orthodontic therapy can be combined with both medical and surgical therapy. The orthodontic approach plays a significant role in cases with a narrow palate, mandibular hypoplasia or retruded mandible. The aim of the treatment is to enlarge the volume of the hard palate by means of a fixed orthodontic appliance called a rapid palatal expander, which acts actively by dislocating the median palatine suture for 3–4 months [21]. This can also be subsequently combined with an intraoral thruster, which allows the advancement of the mandible when it is not correctly positioned due to dental malocclusions.

### 6.4. Myofunctional Therapy

This is a rehabilitation intervention recommended in cases not completely resolved after AT or orthodontic treatment [22].

### 6.5. Ventilation Therapy with Positive Pressure Devices

The indication that surgery has failed occurs when it is contraindicated and when consent to surgery is refused [23,24,25]. It is a non-invasive technique that allows CPAP or BiPAP to be delivered through a mask into the airways. The aim of this treatment is to ensure patency of the upper airway during sleep. Titration of the device should be carried out by performing cardio-respiratory monitoring or PSG and, if possible, also continuous CO_2_ measurement. Therapy with positive pressure devices is usually well tolerated (about 80% of cases). In the case of uncooperative children, several attempts should be made to improve compliance before moving on to an alternative treatment option. In general, multidisciplinary post-treatment follow-up involving the paediatrician of choice and the hospital centre team is essential to assess symptom improvement or to identify residual and/or persistent disorders.

## 7. Pathways Developed in Emilia-Romagna Region, Italy

In agreement with the European Respiratory Society recommendations) [2,3], the multidisciplinary group of experts on paediatric OSA from Emilia-Romagna Region, Italy, distinguished two different ‘step’ pathways, depending on the age group considered: under or over two years.

### 7.1. Stepwise Management of OSA in Patients ≤ 23 Months Old

***STEP 1***

**Identification of individuals at risk of OSA**

Presence of one or more of the following:

1. Symptoms of upper respiratory tract obstruction (i.e., snoring, apnoea, insomnia), history of apparent life threatening events (ALTE), with possible secondary role of gastroesophageal reflux and prematurity;
2. Growth retardation;
3. Feedback on adenoid or, less frequently, tonsillar hypertrophy; nasal obstruction; laryngeal obstruction; syndromic craniosynostosis and/or hypoplasia of the middle third of the face; cleft palate; mandibular hypoplasia (e.g., Pierre Robin sequence); neuromuscular disorders; complex pathologies (e.g., achondroplasia, Down syndrome);
4. Endoscopic finding of upper airway abnormalities.

***STEP 2***

**Searching for comorbidities** (pulmonary hypertension and pulmonary heart, growth retardation of the body, behavioural problems) and coexisting conditions (i.e., eating disorders).

***STEP 3***

**Diagnosis and assessment of the severity of OSA with the involvement of a multidisciplinary team** (involving paediatric pulmonologist, otorhinolaryngologist, paediatric neuropsychiatrist/neurologist, orthodontist, maxillofacial surgeon and others, depending on the individual case).

Use of diagnostic tools: PSG (gold standard) or, alternatively, polygraphy or nocturnal pulse oximetry

Cut-off values for defining OSA and its severity:

1. AHI < 1 and desaturations > 3% max 2.2/h in healthy children
2. Mild OSA: AHI 1–5 episodes/h
3. Moderate OSA: AHI > 5–10 episodes/h
4. Severe OSA: AHI > 10 episodes/h

***STEP 4***

**Treatment of OSA** if pathological PSG, polygraphy or pulse oximetry are associated with snoring, oral breathing or tachypnoea, ALTE, growth restriction, tonsillar hypertrophy, laryngeal or nasal obstruction, cleft lip and palate, syndromes with craniostenosis or facial hypoplasia, neuromuscular disorders. Treatment becomes a priority in cases of achondroplasia, Beck-Wiedemann syndrome, Chiari malformation, down syndrome, mucopolysaccharidosis or Prader-Willi syndrome.

***STEP 5***

**Individualised approach of the multidisciplinary team based on aetiology, severity of OSA and comorbidities.**

Examples: use of non-invasive ventilation such as CPAP, surgical correction in case of coanal atresia, severe laryngomalacia, mandibular hypoplasia, craniostenosis, need for tracheostomy in case of severe obstruction.

***STEP 6***

**Follow-up and management of persistent OSA**

After AT, OSA may recur usually after four to six months, sometimes requiring further adenoidectomy. In CPAP patients it is necessary to re-evaluate nocturnal saturation every 2–4 months during the first 12 months of treatment and then every 6 months (interface size, pressures, confirmation of necessity). In patients with BiPAP (e.g., neuromuscular patients) it is necessary to re-evaluate saturation every year. After supraglottoplasty it is necessary to re-evaluate the patient after one to six months. In children with Pierre Robin sequence hormone, therapy and ventilation with positive pressure devices are recommended and in patients operated with mandibular distraction it is necessary a frequent follow-up to evaluate the effectiveness of the intervention [26].

**Suggested cases in selected clinical conditions**

- Pierre Robin syndrome: if the McGill score is <2 and there is no pharyngeal collapse at endoscopy, try the prone position; if the score is >2 or AHI > 10 consider orthodontic appliance, nCPAP (if AHI > 10/h) or glossopexy. Collaboration with the maxillofacial surgeon is essential for an integrated approach to the pathology and to decide on the best timing for mandibular distraction and tracheostomy placement.
- Achondroplasia: AT, nCPAP.
- Down syndrome: AT, nCPAP, supraglottoplasty.
- Prader Willi syndrome: AT, oxygen therapy if central apnoea.
- Mucopolysaccharidosis: AT, nCPAP, replacement therapy.

### 7.2. Stepwise Management of OSA in Patients 2–18 Years Old

***STEP 1***

**Identification of individuals at risk of respiratory or sleep-related disorders**

Presence of one or more of the following:

- Symptoms of upper respiratory tract obstruction (e.g., snoring, apnoea, insomnia, shortness of breath);
- Feedback from:
  ✓ Tonsillar hypertrophy;
  ✓ Obesity
  ✓ Hypoplasia of the middle third of the face
  ✓ Mandibular hypoplasia (e.g., Pierre Robin sequence);
  ✓ Neuromuscular disorders;
  ✓ Complex pathologies (i.e., Prader Willi syndrome, Down syndrome); prematurity
  ✓ Familiarity with obstructive sleep disorder;

- Endoscopic finding of upper airway abnormalities.

***STEP 2***

**Search for comorbidities** (e.g., pulmonary hypertension and pulmonary heart, daytime sleepiness, hyperactivity, learning disabilities, behavioural problems) and coexisting conditions (e.g., enuresis, poor growth, feeding disorders, recurrent wheezing, metabolic syndrome).

***STEP 3***

Identification of predictors of persistent obstructive sleep disorder:

- Obesity;
- Male;
- AHI > 5/h;
- African ethnic group;
- Untreated tonsillar hypertrophy.

***STEP 4***

**Objective diagnosis of obstructive sleep breathing disorders and their severity**

PSG (gold standard) or, alternatively, polygraphy. Nocturnal pulse oximetry when the above are not available, although taking into account the limitations mentioned above.

Definition of OSA 1: symptoms of obstructive sleep disorder in combination with AHI

> 2/h or obstructive apnoea index > 1/h and/or adenotonsillar hypertrophy.

Definition of OSA 2: symptoms of obstructive sleep disorder in combination with AHI

> 1/h and/or adenotonsillar hypertrophy.

Cut-off values for defining obstructive sleep breathing disorders and their severity:

- In children without sleep disturbance, it can be valued inside normal range AHI up to 2.5/h between 2 and 6 years and up to 2.1 from 6 to 18 years
- Mild OSA: AHI 1–5 episodes/h
- Moderate OSA: AHI > 5–10 episodes/h
- Severe OSA: AHI > 10 episodes/h

***STEP 5***

**Possible indications for the treatment of obstructive sleep disorder**

Cut-off values for the treatment of paediatric patients with OSA are reported below:

- AHI > 5/h
- AHI 1–5/h if cardiovascular or nervous morbidities, enuresis, growth retardation, risk factors for persistent obstructive sleep disorders
- Positive pulse oximetry + positive questionnaires

Treatment becomes a priority in cases of achondroplasia, Chiari malformation, Down’s syndrome, mucopolysaccharidosis, Prader Willi syndrome.

***STEP 6***

**Individualised approach of the multidisciplinary team based on aetiology, severity of OSA and comorbidities**

Possible approached based on comorbidities are reported below:

- Weight loss if obese;
- Nasal steroids and/or montelukast;
- AT;
- Maxillary expander or orthodontic appliances;
- CPAP or BiPAP if hypoventilation;
- Maxillofacial surgery;
- Tracheostomy.

***STEP 7***

**Follow-up and management of persistent OSA**

In the follow-up of paediatric patients with OSA, clinical and polysomnographic control will always be anticipated in case of recurrence of nocturnal symptoms (in particular snoring and apnoeas reported by parents) and daytime symptoms. The procedures to be followed are outlined below:

- Six weeks after AT, repeat PSG;
- After 12 weeks of treatment with nasal steroids and/or Montelukast repeat PSG;
- After 12 months of maxillary expansion and after 6 months of orthodontic appliances, repeat PSG;
- In case of CPAP or BiPAP, repeat PSG annually.

The differences and similarities in the diagnosis and management of sleep-related obstructive respiratory disorders in young children (0–23 months) and older patients (2–18 years) are shown in Table 2.

## 8. Conclusions

The flow-chart of the diagnostic-therapeutic pathway for the management of OSA in paediatrics, regardless of the patient’s age, can be divided into the three phases (Table 3).

In most cases, this pathway can be carried out by the primary care paediatrician, who represents the first filter for approaching the problem and who must be informed of the various phases of the diagnostic-therapeutic pathway. For this reason, it is essential that the primary care paediatrician receives adequate training on how to formulate the diagnostic suspicion of OSA and on what criteria to use to select patients to be sent to the hospital centre. The relationship between the paediatrician of the patient and her/his parents must see a synergy of behaviour between the various players in order to avoid uncertainty about the diagnostic and therapeutic decisions to be taken. The patient and her/his parents must see a synergy of behaviour between the various players in order to avoid uncertainty about the diagnostic and therapeutic decisions to be taken. It is particularly important to emphasise the follow-up phase following any treatment carried out, in which scheduled checks by hospital centres are necessary and in which the primary care paediatrician must remain the main reference point for families. The definition and evaluation of the organizational, process and outcome indicators of the developed flow chart and the impact of its implementation will remain fundamental.

This protocol has been developed by the multidisciplinary contribution of experts belonging to different specializations and represents, in our opinion, the most complete and up-to-date collection of recommendations regarding OSA management in paediatric age. The application of uniform and shared protocols aims to improve clinical practice, through the standardization of diagnostic procedures and therapeutic approaches. In order to overcome barriers or hurdles, a strong educational activity associated with tools such as audit and feedback as a moment of “self-analysis” of a health organization, focus groups that give space for discussion, and the support of the political decision-maker are key elements for the success of the implementations of these recommendations.

## Figures and Tables

**Table 1:** Cognitive and behavioural deficits associated with sleep disorders in paediatric patients with OSA.

Cognitive Deficits	Behavioral Deficits
Intellectual deficits, verbalization disorder, poor vocabulary, behavioural anormalities	Hyperactivity/attention deficit hyperactivity disorder
Learning deficits	Somatisation
Poor school performance	Aggression and social problems
Poor impulse control	Excessive daytime sleepiness
Attention deficits	Anxiety

From Marcus CL et al. (2012), modified [7].

**Table 2:** Differences and similarities in the diagnosis and management of sleep-related obstructive respiratory disorders in young children (0–23 months) and older patients (2–18 years).

Diagnosis	Patients 0–23 Months	Patients 2–18 Years
Symptoms of upper airway obstruction present in both wakefulness and sleep	Yes	No
Adenotonsillar hypertrophy and obesity as a cause of sleep-related obstructive respiratory disorders	Yes, but uncommon	Yes
Syndromes, congenital anomalies as a cause of sleep-related obstructive respiratory disorders	Yes	Yes
Feeding difficulties and poor growth can coexist with OSA	Yes	No
Pulmonary hypertension can complicate OSA	Yes	Yes
Polysomnography as the gold standard for OSA	Yes	Yes
Endoscopy useful for assessing upper airway collapse	Yes	No
Management	Yes	Yes
Adenotonsillectomy is the most useful treatment	Yes	Yes
Non-invasive ventilation is often used as a first treatment for dynamic airway collapse	Yes	No
Effective orthodontic appliances in cases of OSA with retrognathia and Malocclusion	No	Yes
Patients with complex conditions to be treated as a priority	Yes	Yes
Follow-up after surgery should detect persistent OSA	Yes	Yes
Patients on non-invasive ventilation undergo annual nocturnal saturation monitoring	Yes	Yes

**Table 3:** Flow chart for the classification of OSA in paediatric age.

Phase	Actors	Actions	Tools
Phase 1	Primary care Paediatricians clinic	Medical history/ objective examination	Pediatric sleep questionnaire Teenager STOP BANG
Phase 2	First level OSA outpatient clinic	ENT examination Rhinofibroscopy Pulse oximetry/polysomnography Specialist surgical examination Medical therapy Orthodontic therapy Myofunctional therapy Diet	
Phase 3	2nd level OSA outpatient clinic	Diagnosis (pulse oximetry, polygraphy, polysomnography, DISE) Therapy (medical and ventilatory) follow-up	Surgical therapy (regionally licensed hospital for paediatric surgery in children 2 years of age/presence of resuscitation paediatric)

## Data Availability

All of the data are included in the manuscript.

## Supplementary Materials

The following are available online at https://www.mdpi.com/article/10.3390/life12050739/s1, Supplementary material S1: Pediatric Sleep Questionnaire. Supplementary material S2: Teenager STOP-BANG.
