# Supplementary material for: Diagnostic and Therapeutic Approach to Children and Adolescents with Obstructive Sleep Apnea Syndrome (OSA): Recommendations in Emilia-Romagna Region, Italy
Source: Life (Basel). 2022 May 16;12(5):739. doi: 10.3390/life12050739 (PMC9146195; doi:10.3390/life12050739)
Supplement: Supplementary file 1 [file life-12-00739-s001.zip › Supplementary Material S1.pdf]

Supplementary Material S1

Surname			Name
**PAEDIATRIC SLEEP QUESTIONNAIRE- SCREENING QUESTIONNAIRE FOR OSA**			
**1) The child during sleep:**			
Always snores	YES	NO	NOT SO
She snores at least half the night	YES	NO	NOT SO
Snoring noisily	YES	NO	NOT SO
Breathing 'heavily' or noisily	YES	NO	NOT SO
Presents breathing difficulties	YES	NO	NOT SO
**2) The child stops breathing during sleep.**	YES	NO	NOT SO
**3) The child:**			
Breathe with your mouth open during the day	YES	NO	NOT SO
Presents with dry mouth on awakening	YES	NO	NOT SO
Presents enuresis	YES	NO	NOT SO
**4) The child:**	YES	NO	NOT SO
He wakes up tired	YES	NO	NOT SO
Appears sleepy during the day	YES	NO	NOT SO
**5) Has the teacher ever told you that you appear drowsy:**	YES	NO	NOT SO
**6) Wakes up with difficulty in the morning:**	YES	NO	NOT SO
**7) Wakes up complaining of headache:**	YES	NO	NOT SO
**8) It has shown a slowdown in growth:**	YES	NO	NOT SO
**9) He is overweight:**	YES	NO	NOT SO
**10) Often the child:**	YES	NO	NOT SO
Seems not to listen	YES	NO	NOT SO
Easily distracted	YES	NO	NOT SO
He has difficulty in doing his homework	YES	NO	NOT SO
He is agitated when sitting	YES	NO	NOT SO
Interrupts adult conversations	YES	NO	NOT SO
He is so agitated that he seems 'spring-loaded'.	YES	NO	NOT SO
**Score:** YES = 1 NO = 0<br>**Ratio of Positive Responses/Total Responses** (22) > 0.33 = Out of Standard			

Chervin R.D et al 2000 [10].
