# Supplementary material for: Diagnostic and Therapeutic Approach to Children and Adolescents with Obstructive Sleep Apnea Syndrome (OSA): Recommendations in Emilia-Romagna Region, Italy
Source: Life (Basel). 2022 May 16;12(5):739. doi: 10.3390/life12050739 (PMC9146195; doi:10.3390/life12050739)
Supplement: Supplementary file 1 [file life-12-00739-s001.zip › Supplementary Material S2.pdf]

Supplementary material S2

Surname				Name		
**Teenager**<br>**STOP-BANG**						
Snoring (How often do you snore loudly?)	I do not know	NEVER	Rarely	Occasionally	Frequently	Always
Tired (Feels tired during the day (daytime tiredness)	I do not know	NEVER	Rarely	Occasionally	Frequently	Always
Observed apnoea (How often did you observe pauses in breathing in their sleep)	I do not know	NEVER	Rarely	Occasionally	Frequently	Always
Pressure (systolic or diastolic p.p. >= 95 pecentile for age and height)	I do not know	NEVER	Rarely	Occasionally	Frequently	Always
BMI (greater than or equal to the 95 percetiles for age and height)	S<br>I			N<br>O		
Academic problems (learning/scholastic problems)	I do not know	NEVER	Rarely	Occasionally	Frequently	Always
Neck circumference (neck circumconference >= of 95 percentile for age and height)	S<br>I			N<br>O		
Gender (Male)	S<br>I			N<br>O		
Score:<br>Response Ratio/Total answers (8) ><br>(Pathological>= 4)						

Combs D et al 2015 [11]
